# Fluconazole‐Induced Electrolyte Disturbances and Cardiac Toxicity in a CRRT‐Dependent Patient With Acute Renal Failure

**DOI:** 10.1002/ccr3.71292

**Published:** 2025-10-22

**Authors:** Fuxia Jian, YuShan Zhang, Shili Zhong, Zhen Wang, Cui Shang

**Affiliations:** ^1^ Department of Intensive Care Medicine Army Medical Center of PLA Chongqing China

**Keywords:** arrhythmia, fluconazole, hyperkalemia, QT extension, renal failure

## Abstract

Fluconazole is a commonly used antifungal drug in clinical practice. Both hyperkalemia and QT interval prolongation are life‐threatening complications of fluconazole. Our report warns clinicians to pay attention to the cardiotoxicity and electrolyte disorders of fluconazole when using it to avoid serious consequences.

## 1 Introduction

Fluconazole is a triazole antifungal drug. Its main mechanism of action is to highly selectively inhibit 14α‐lanosterol demethylation mediated by fungal cytochrome P450 enzymes, thereby suppressing the biosynthesis of fungal ergosterol [1]. Common adverse reactions of fluconazole include nausea, vomiting, headache, rash, reduction of three blood cell lines, hypercholesterolemia, hypokalemia (with a low incidence), and myalgia, etc. However, its adverse reaction of causing hyperkalemia has rarely been reported [2, 3]. Most of fluconazole is excreted through the kidneys, although approximately 11% seems to be metabolized in the liver [4, 5]. According to current treatment guidelines, for patients with renal failure undergoing continuous CRRT, the loading dose of fluconazole is 6–12 mg/kg, and the maintenance dose is 6 mg/kg [5]. Arrhythmias are very common in critically ill patients in the intensive care unit. However, there is currently no literature reporting the occurrence of hyperkalemia combined with first‐degree atrioventricular block and long QT interval in renal failure patients undergoing CRRT during fluconazole treatment. This case is the first report.

## 2 Case History

The patient was an 83‐year‐old elderly male who underwent partial hepatectomy and cholecystectomy due to liver cancer. Seventeen days after the surgery, he developed sepsis and septic shock. On the 23rd day after the operation, the patient had a persistent high fever, a drop in blood pressure, and an increased heart rate, which were considered septic shock. Given that the patient had a history of subtotal gastrectomy, an enhanced abdominal CT scan was performed. It was highly suspected that the patient had a gastrointestinal perforation. Therefore, the patient underwent an emergency exploratory laparotomy and small intestine repair.

After the surgery, the patient experienced recurrent fever and elevated infection markers. He also developed multiple organ failures involving the heart, kidneys, lungs, and liver. For further treatment, the patient was transferred to our hospital. On the day of admission, the patient's oxygenation index was 150 mmHg, BNP was elevated, there was continuous anuria, and levels of blood urea nitrogen and creatinine were abnormal. Additionally, the patient had recurrent arrhythmias. Subsequently, the patient was intubated and placed on invasive mechanical ventilation. Blood cultures were taken from the PICC catheter and femoral vein catheter. After replacing the hemofiltration catheter, continuous CRRT treatment was initiated.

**FIGURE 1:**
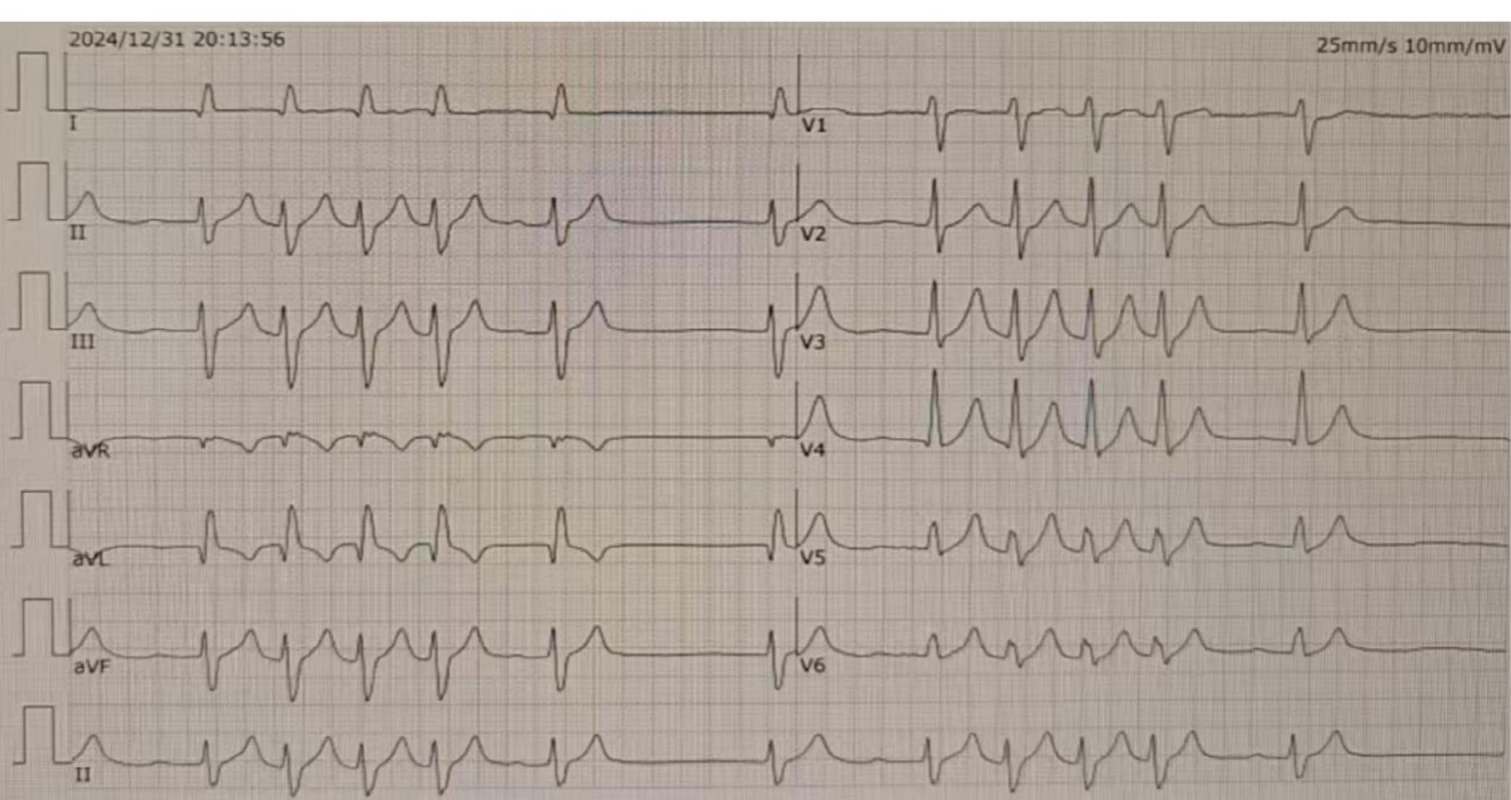
Electrocardiogram of a patient after fluconazole.

The culture of the patient's abdominal drainage fluid obtained outside the hospital showed *Candida albicans* , which was sensitive to fluconazole. High‐throughput sequencing of the patient's ascites and blood also confirmed the presence of *Candida albicans* . The patient was given an initial dose of 600 mg of fluconazole, followed by a maintenance dose of 400 mg. Two hours after starting fluconazole treatment during continuous CRRT, the patient developed hyperkalemia, with a blood potassium level of 7.0 mmol/L. At the same time, blood lactate increased to 13.2 mmol/L, and the patient developed first‐degree atrioventricular block and a prolonged QT interval (Figure 1).

## 3 Conclusion and Results

Since no potassium chloride was added to the replacement fluid, rapid medical potassium‐lowering treatment was immediately administered. Subsequently, the patient's blood potassium level dropped to the normal range, and blood lactate gradually decreased. Later in the treatment, caspofungin was used to replace fluconazole, and the patient did not experience hyperkalemia again.

## 4 Discussion

In this case, the patient did not receive any potassium‐containing fluids during continuous CRRT treatment. Hyperkalemia developed after fluconazole administration and did not recur following drug discontinuation. According to the instructions of fluconazole, its induction of hyperkalemia is an extremely rare complication. In 2015, Ozlem Elkiran reported a case of hyperkalemia induced by fluconazole in a critically ill infant [6]. Current research suggests that there are three reasons for hyperkalemia after the use of fluconazole: (1) Impact on adrenal cortex function: Fluconazole can inhibit the steroid synthesis of the adrenal cortex, leading to a decrease in aldosterone secretion. Aldosterone plays a key role in regulating potassium ion excretion, and its decrease may lead to potassium ion retention. (2) Drug interactions: Fluconazole may interact with other drugs (e.g., ACEI, ARB, levofloxacin), further increasing the risk of potassium ion retention. (3) Alteration of the distribution of potassium ions inside and outside the cell: In some cases, fluconazole may affect the function of ion channels in the cell membrane, causing potassium ions to be released from the inside to the outside of the cell [7, 8, 9]. Although this patient had acute renal failure due to severe infection, the serum potassium level fluctuated within the normal range in the 2 days before admission without dialysis. Hyperkalemia occurred within 6 h after using fluconazole, which may be related to the patient's acute renal failure and impaired adrenal cortex function.

Upon admission, the patient's electrocardiogram (ECG) did not show a long QT interval, and the serum potassium level in the blood gas analysis was within the normal range. The echocardiogram showed ventricular septal thickening. Hyperkalemia, first‐degree atrioventricular block, and a long QT interval occurred within 24 h after using fluconazole, which supports the view that fluconazole‐induced these conditions. The prolongation of the QT interval can be divided into genetic and acquired types. In this case, the patient had no history of arrhythmia, and there was no history of QT prolongation during the off‐hospital treatment. We considered that the patient had acquired QT interval prolongation. Currently, studies have reported that fluconazole can cause QT interval prolongation and torsades de pointes [9]. Current research suggests that fluconazole leads to QT interval prolongation in the following situations: (1) Inhibition of the hERG potassium channel, which is responsible for the rapid delayed rectifier potassium current (IKr) during cardiac repolarization. Inhibiting the hERG channel reduces potassium ion efflux, prolongs the action potential duration of cardiomyocytes, and thus leads to QT interval prolongation. (2) Effects on other ion channels. Besides the hERG channel, fluconazole may also affect other ion channels (such as sodium and calcium channels), further interfering with cardiac electrical activity and increasing the risk of QT interval prolongation [7, 10, 11]. (3) Fluconazole is a strong inhibitor of the CYP3A4 enzyme and may affect the blood drug concentrations of other drugs metabolized by this enzyme (e.g., certain anti‐arrhythmic drugs and antipsychotics), increasing the risk of QT interval prolongation. (4) The risk of QT interval prolongation is higher when fluconazole is used at high doses or for a long time. In 2009, Chakravarty C reported a case of a patient with diabetic ketoacidosis who developed torsades de pointes ventricular tachycardia under the conditions of hypokalemia and hypocalcemia, resulting in nine cardiac arrests secondary to intravenous fluconazole administration [12].

In this patient, after detecting hyperkalemia and QT prolongation, the drug was promptly discontinued, and fortunately, the patient did not develop severe arrhythmias such as ventricular tachycardia or cardiac arrest. Most critically ill fungal‐infected patients have multiple organ failure and are prone to atrial fibrillation. Therefore, fluconazole and amiodarone are often used in combination in clinical practice. It is particularly noteworthy that the combination of these two drugs can easily induce life‐threatening arrhythmias [13]. Research reports have found that fluconazole can induce arrhythmias not only when combined with amiodarone but also when combined with drugs such as amitriptyline, ciprofloxacin, and levofloxacin. It is necessary to carefully observe for the occurrence of arrhythmias and, if necessary, continuously monitor the ECG [14, 15, 16, 17]. Due to reports of fluconazole‐induced fatal arrhythmias, clinicians often pay less attention to related non‐fatal arrhythmias. Kumi Tamura encountered a case of fluconazole‐induced frequent ventricular premature beats during the treatment of HIV‐related pulmonary cryptococcosis. This also warns us that when using fluconazole clinically, if a patient suddenly develops frequent ventricular premature beats, we should be vigilant against it being a side effect of fluconazole [18].

In conclusion, our report emphasizes that there is still a risk of hyperkalemia and arrhythmia in patients undergoing continuous CRRT treatment when using fluconazole. Clinicians must closely monitor ECGs and electrolytes when fluconazole is used alone or in combination with other drugs to prevent life‐threatening arrhythmias and cardiac arrest.

## Author Contributions

**Fuxia Jian:** conceptualization, writing – original draft, writing – review and editing. **YuShan Zhang:** investigation, visualization, writing – original draft. **Shili Zhong:** conceptualization, data curation, software, validation. **Zhen Wang:** investigation, software, supervision, visualization. **Cui Shang:** conceptualization, writing – original draft, writing – review and editing.

## Ethics Statement

This study is based on the Declaration of Helsinki and approved by the Ethics Committee of the Army Medical Center of PLA.

## Consent

Written informed consent was approved by all authors. Written informed consent was also obtained from the patient to publish this report in accordance with the journal's patient consent policy.

## Conflicts of Interest

The authors declare no conflicts of interest.

## Data Availability

The original contributions presented in the study are included in the article/Supporting Information; further inquiries can be directed to the corresponding author.
